# Sleep problems and their interaction with physical activity and fatigue in hematological cancer patients during onset of high dose chemotherapy

**DOI:** 10.1007/s00520-021-06377-5

**Published:** 2021-07-10

**Authors:** Lucia Castelli, Thomas Elter, Florian Wolf, Matthew Watson, Alexander Schenk, Karen Steindorf, Wilhelm Bloch, Michael Hallek, Niklas Joisten, Philipp Zimmer

**Affiliations:** 1 Department of Biomedical Sciences for Health, University of Milan, Via Giuseppe Colombo 71, 20133 Milan, Italy; 2 Department I of Internal Medicine, Center for Integrated Oncology Aachen Bonn Cologne Duesseldorf, University of Cologne, Kerpener Straße 62, 50937 Cologne, Germany; 3 Department of Molecular and Cellular Sports Medicine, Institute of Cardiovascular Research and Sports Medicine, German Sport University Cologne, Am Sportpark Müngersdorf 6, 50933 Cologne, Germany; 4 Institute of Psychology, German Sport University Cologne, Am Sportpark Müngersdorf 6, 50933 Cologne, Germany; 5 Division for Performance and Health (Sports Medicine), Institute for Sport and Sport Science, TU Dortmund University, Otto-Hahn-Straße, 344227 Dortmund, Germany; 6 Division of Physical Activity, Prevention and Cancer, German Cancer Research Center (DKFZ), National Center for Tumor Diseases (NCT) Heidelberg, Im Neuenheimer Feld 460, 69120 Heidelberg, Germany

**Keywords:** Sleep, Physical activity, Fatigue, Hematological cancer, Leukemia

## Abstract

**Purpose:**

Sleep problems reported by hematological cancer patients are usually linked to higher levels of cancer-related fatigue. Although the awareness of sleep problems in solid cancer patients is rising, there has been less attention to the issue in hematological cancer patients.

The present study assesses the differences in sleep by comparing physical activity and fatigue levels among hematological cancer patients during the onset of chemotherapy. Furthermore, it investigates the relationship between sleep, physical activity, and fatigue through mediation analysis.

**Methods:**

The recruited sample consists of 58 newly diagnosed hematological cancer patients (47.1 ± 15.4 yrs; 51.7% males). Subjects completed questionnaires assessing sleep (PSQI), physical activity (visual analogue scale), fatigue (MFI-20), anxiety, depression (HADS), and quality of life (EORTC QLQ-C30) within two weeks from starting treatment.

**Results:**

The sample reported more sleep problems in comparison to the German population norm. The classification as good (ca 25%) or bad sleepers (ca 75%) showed less frequent physical activity (*p* = .04), higher fatigue (*p* = .032), anxiety (*p* = .003), depression (*p* = .011) and pain (*p* = .011) in bad sleepers. The mediation analysis revealed significant indirect effects of sleep on fatigue through physical activity habits.

**Conclusions:**

This study highlights the combined action of sleep problems and physical activity on fatigue during the onset of induction chemotherapy. These two parameters could represent meaningful intervention targets to improve a patient’s status during chemotherapy.

**Trial registration:**

The study was registered on the WHO trial register (DRKS00007824).

## Introduction

Sleep problems are among the most frequently reported complaints in cancer patients across all stages of the disease (i.e., before, during, and after treatment) [1, 2]. This issue has received research attention in patients with solid tumors but has rarely been investigated in hematological cancer patients [3–5].

Rather than being considered an independent issue, sleep problems in hematological cancer patients are considered to be a consequence of symptoms such as cancer-related fatigue [5]. Indeed, previous assessment of sleep problems emerged in part while evaluating other problems linked to hematological cancer, describing it as one of the five symptoms affecting leukemia patients [6]. Sleep problems in cancer patients are influenced by several factors, including hospitalization in an unfamiliar environment, a situation that adds concerns compared to receiving ambulatory therapy. Interestingly, sleep problems have been associated with lower survival chances [4, 7] and overall quality of life [5]. In regard to alleviating impaired sleep, studies have demonstrated how regular physical activity and body-mind disciplines can improve sleep quality [8–12].

Less time spent in physical activity is another complaint described by both solid and hematological cancer patients [13]. Indeed, patients affected by leukemia are reported to be less physically active even before the diagnosis [14]. As it has been shown that maintaining and improving one’s physical capabilities can reduce several side effects of the disease and its treatment, an increase in physical activity levels is recommended before, during, and after medical therapy [15, 16]. However, investigations on both sleep and physical activity are mainly conducted during and after medical treatments, whereas studies during the onset of medical therapies are lacking.

Sleep problems and reduced physical activity are two factors that strongly link to the sensation of cancer-related fatigue in leukemia patients, which can interfere with chemotherapy toleration and survival outcomes [16, 17]. Cancer-related fatigue in leukemia patients is a multifactorial symptom that is often already present at the time of diagnosis and during the onset of therapies [18, 19]. In addition to the adverse effects of therapies, also muscle dysfunction could increase cancer-related fatigue perception in hematological cancer patients. Reduced physical activity can augment cancer-related fatigue as it contributes to progressive deconditioning of muscle and strength [20]. Together, cancer-related fatigue and reduced physical activity act in a vicious circle, in which higher cancer-related fatigue reduces one’s physical activity while lower levels of physical activity reciprocally increase one’s sensation of cancer-related fatigue [21].

In summary, leukemia patients are often identified as fatigued subjects that report sleep and physical activity impairments at the time of diagnosis [16]. To date, no studies have tried to assess how sleep, physical activity, and fatigue interact and interfere with each other during the onset of chemotherapy. However, such studies are necessary to better understand the relationship between these factors and to develop efficient supportive treatments to reduce this complex of symptoms.

In view of this, the present study considers individuals with hematological malignancies during the onset of high-dose chemotherapy. It focuses on the role of sleep on physical activity and fatigue levels between those classified as good or bad sleepers. Additionally, a mediation model will be used to investigate interactions between sleep, physical activity, and fatigue.

## Materials and methods

### Study design and procedure

The present study presents baseline evaluation data collected as part of a more extensive study between May 2017 and February 2019 [22]. In brief, patients diagnosed with hematological cancer were recruited to participate on a voluntary basis at the Department I of Internal Medicine, Center for Integrated Oncology Cologne/Bonn at the University of Cologne. As soon as possible after the time of diagnosis (in general, two days after the diagnosis), patients were informed about the study aim and invited to participate. Upon deciding to participate and within two weeks after treatments onset, patients completed a pen and paper informed consent form and questionnaires to evaluate sleep, physical activity, fatigue, anxiety, depression, and quality of life.

The study was conducted according to the latest version of the Declaration of Helsinki and received approval by the University Hospital of Cologne's Ethical Committee. The study was registered on the WHO trial register (DRKS00007824).

### Participants

Initially, 72 participants were included in the study; however, datasets of 58 participants were available with all necessary outcomes data for the present investigation.

Inclusion criteria for the present analysis were: age > 18 years; diagnosis of acute myeloid leukemia (AML) (excluding AML subtype M3), acute lymphoblastic leukemia (ALL), non-Hodgkin lymphoma (NHL); no metastases in the central nervous system; no neurodegenerative disease diagnosis (e.g., dementia or Morbus Parkinson); questionnaires must be completed within two weeks after onset of high dose chemotherapy.

### Measurements

#### Anthropometric and descriptive data

The height and weight of each participant were determined to calculate their BMI (kg/m^2^). Subsequently, participants completed a form pertaining to general characteristics (age, smoking habits, occupational status, education years) and their respective pathology (diagnosis and the number of comorbidities).

#### Questionnaires

*Pittsburgh Sleep Quality Index*

To assess sleep, we utilized the German version of the Pittsburgh Sleep Quality Index (PSQI) [23, 24]. This index, based on 19 items’ results, is a retrospective self-report questionnaire that assesses sleep over the 30 days prior to completion. The total score ranges between 0 and 21, where a lower score indicates lower impaired sleep. The cut-off value of 5 differentiates between *good sleepers* (0–5) and *bad sleepers* (6–21).

*Physical Activity Scale*

Daily physical activity over the prior four weeks was assessed with the Physical Activity Scale (PAS). The PAS consists of a visual scale ranging from 0 (inactive) to 10 (very active). Thus, the higher the PAS values, the higher the level of physical activity.

*Multidimensional Fatigue Inventory*

The German version of the Multidimensional Fatigue Inventory (MFI) was used to assess fatigue [25]. The MFI is a self-report questionnaire, lying on 20 items, in which general fatigue over the days prior to completion is reported. Higher values indicate a higher level of fatigue [26].

*Hospital Anxiety and Depression Scale*

The Hospital Anxiety and Depression Scale (HADS) German version was used to assess anxiety and depression through 14 items [27, 28]. The HADS outcomes consist of a total, anxiety and depression score, in which the higher the value, the higher the level of anxiety and depression.

*European Organization for Research and Treatment of Cancer – Quality of life*

The European Organisation for Research and Treatment of Cancer – Quality of life questionnaire (EORTC QLQ-C30) lies on 15 items and was used to assess quality of life [29, 30]. This questionnaire evaluates global health status, function roles, and symptoms during the week prior to completion. High global health and function role scores represent a better quality of life and a healthy functioning level. A high score for symptoms signifies an increased level of symptomatology or problems [31].

### Statistical analysis

Statistical analyses were carried out using the IBM Statistical Package for the Social Sciences—SPSS Statistics version 26 (IBM Corp. Released 2019. IBM SPSS Statistics for Windows, Armonk, NY: IBM Corp).

We calculated the mean, standard deviation, and numbers or percentage for each continuous and categorical variable, respectively. The assumption of normality for each continuous variable was verified with the *Shapiro–Wilk test and Kurtosis and Skewness* assessments. In line with the first aim of this study, we first assessed if our sample had significantly more sleep problems compared to the general German population matched by sex (*One-sample t-Test*) [24]. Secondly, we categorized subjects as either good or bad sleepers based on PSQI total score. We used analysis of covariance (ANCOVA) to compare physical activity, fatigue, anxiety, depression, and quality of life levels between good and bad sleepers. As covariate days since the onset of chemotherapy were included as a continuous variable. Effect size (*d* Cohen) was calculated to quantify the magnitude of continuous variables. Effect size is interpreted as small (*d* = 0.2), medium (*d* = 0.5), or large (*d* = 0.8) [32].

In line with the second aim of this study, and to identify potential variables for mediation analysis, partial correlation analysis (*Pearson coefficient*) was completed. The mediation analysis was subsequently completed and identified associations between sleep, physical activity, and fatigue.

The PROCESS macro version 3.5 for SPSS was used for the mediation analysis. The PROCESS macro integrates the Sobel test with bootstrapping, making mediation feasible and reliable for non-normally distributed variables [33]. We set 5000 bootstraps and used Model 4 (Simple mediation) of the software. The mediation analysis is structured on an independent variable (X) that is correlated with the dependent variable (Y). The correlation could be mediated by a third variable (M) that acts as a mediator between X and Y. In other words, two subjects who differ by one unit of X report significantly different Y values as a result of the M action. The mediation model is described with direct effects (*c'*) and with two indirect effects (*a'* XM; b' MY), which multiply together to describe the total indirect effects (*ab*). Total indirect effects are statistically significant when upper and lower levels do not include 0. In this study, we hypothesize that sleep problems (X) (PSQI total score) could affect fatigue (Y) (MFI general fatigue) through the action of physical activity (M) (PAS). Also, for this analysis, days since the onset of chemotherapy were included as a continuous covariate. The statistical significance and confidence intervals were set at α ≤ 0.05 and 95%, respectively.

## Results

### Descriptive data

Table 1 shows the descriptive data of the sample. The sample was equally composed of males and females. The mean BMI value corresponded to a categorization of overweight. The most represented diagnosis was AML.

**Table 1:** Descriptive data of the total sample reported as mean ± SD or number and percentage

Variable	Mean ± SD	N (%)
Age (years)	47.1 ± 15.4	
Gender		
Male		30 (51.7)
Female		28 (48.3)
Body mass (kg)	80.5 ± 14.6	
BMI (kg/m^2^)	26.1 ± 3.7	
Normal weight		22 (37.9)
Overweight		29 (50)
Obesity Class I		6 (10.3)
Obesity Class II		1 (1.7)
Education years	11.3 ± 1.8	
Smoking habits		
Yes		13 (22.4)
No		36 (62.1)
n.a		9 (15.5)
Occupational status		
Worker		46 (79.3)
Retired		3 (5.2)
Student		6 (10.4)
Housewife		2 (3.4)
n.a		1 (1.7)
Diagnosis		
AML		42 (72.4)
ALL		11 (19)
NHL		5 (8.6)
Number of comorbidities	1.2 ± 1.5	

*BMI*, Body Max Index; *AML*, Acute Myeloid Leukemia; *ALL*, Acute Lymphoblastic Leukemia; *NHL*, non-Hodgkin lymphoma.

**Fig. 1:**
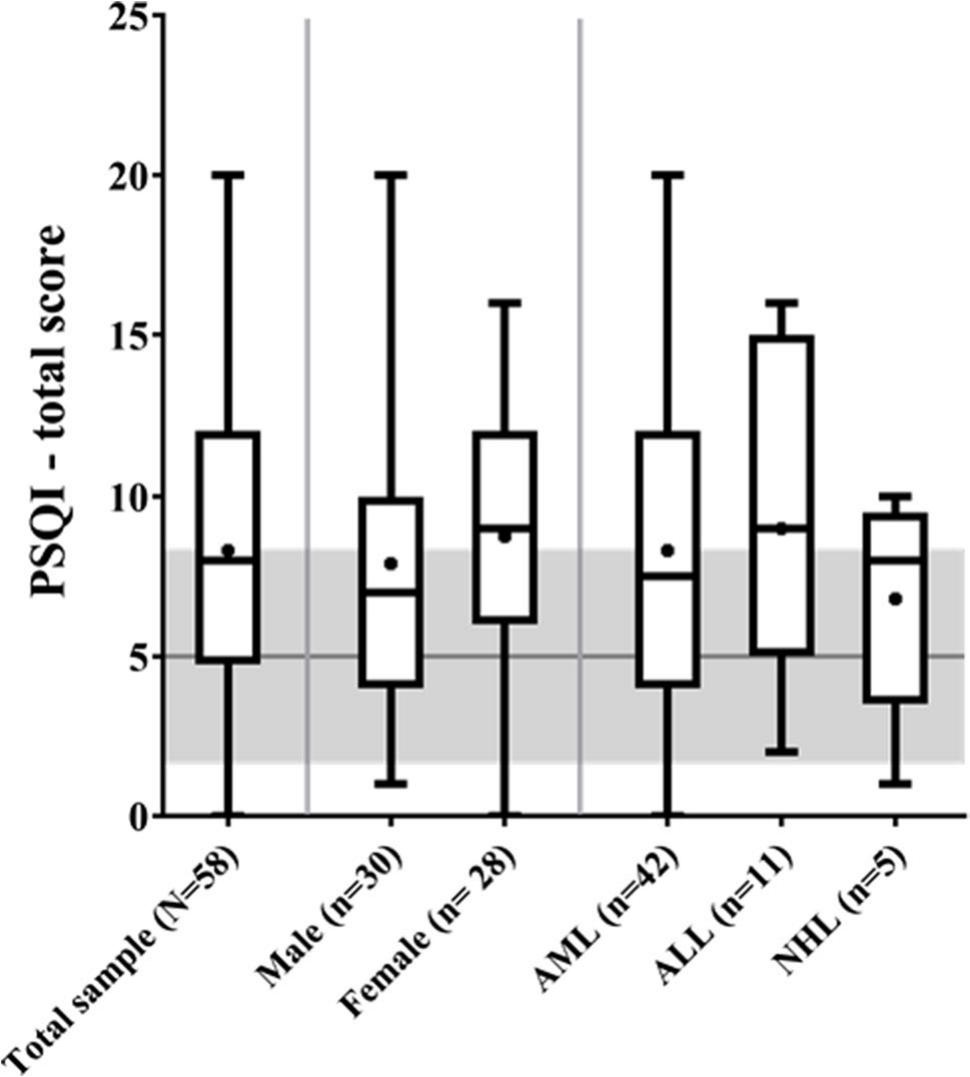
Box-plot representing the entire range of PSQI total values for the whole sample, male, female, AML, ALL, and NHL stratifications. The grey area indicates the general German population PSQI mean and SD; the vertical grey lines separate the total sample from sex and pathology categorizations. PSQI = Pittsburgh Sleep Quality Index; AML = Acute Myeloid Leukemia; ALL = Acute Lymphoblastic Leukemia; NHL = non-Hodgkin lymphoma

The PSQI total score — shown in Table 2 — indicated that the sample was over the cut-off value (5), thus with sleep problems. Indeed, bad sleepers comprised the majority of the sample (n = 43 (74.1%)). As Fig. 1 shows, the entire sample was significantly over mean values for the general German population (5.0 ± 3.4 PSQI final score) (*p* < 0.001) [24], as well as the stratifications by gender (PSQI final score: males, 7.9 ± 4.8, general German males 4.4 ± 3.0, *p* < 0.001; females, 8.9 ± 4.3, general German females 8.9 ± 4.3, *p* = 0.001) and diagnosis (PSQI final score: AML, 8.3 ± 4.6; ALL, 9.0 ± 5.2; NHL, 6.8 ± 3.5; *p* < 0.001,, *p* = 0.02; *p* = 0.04, respectively) [24]. Thus, the present sample reported more sleep problems in comparison to the German population norm.

**Table 2:** Questionnaires results for the total sample (mean ± SD), and comparison data between good and bad sleepers (mean ± SD, p-value, and d-Effect size)

Variable	Mean ± SD	Good sleeper n = 15	Bad sleeper n = 43	*F*	*CI* *(LCI)–(UCI)*	*p*	*d*
PSQI Total score	8.3 ± 4.6	3 ± 1.4	10.2 ± 3.7	27.8	(− 8.9)–(− 5.0)	** < 0.001**	2.2
PAS	4.5 ± 3.0	6.1 ± 2.9	3.9 ± 2.9	3.8	(0.9)–(3.8)	**0.040**	0.8
MFI General fatigue	13.3 ± 3.9	11.3 ± 4.3	14.1 ± 3.5	2.5	(− 5.3)–(− 0.2)	**0.032**	− 0.8
HADS							
Total score	12.5 ± 7.5	6.9 ± 4.7	14.5 ± 7.4	5.6	(− 12.3)–(− 3)	**0.002**	1.1
Anxiety	7.1 ± 4.3	4.0 ± 3.0	8.2 ± 4.2	5.1	(− 6.8)–(− 1.5)	**0.003**	1.1
Depression	5.4 ± 4.2	2.9 ± 2.5	6.3 ± 4.4	3.7	(− 6.1)–(− 0.8)	**0.011**	0.8
EORTC-QOL-30							
Global health status	41.8 ± 27.4	53.3 ± 28.1	37.8 ± 26.3	1.5	(− 2.3)–(33.1)	0.088	0.6
Physical functioning	70.9 ± 25.7	77.3 ± 27.0	68.8 ± 25.2	0.5	(− 9.9)–(25.9)	0.372	0.3
Role functioning	46.4 ± 37.1	62.2 ± 37.0	40.4 ± 35.8	1.9	(− 4.2)–(43.9)	0.103	0.6
Emotional functioning	53.4 ± 27.6	72.8 ± 21.7	46.7 ± 26.4	4.5	(8.4)–(42.6)	**0.004**	1.0
Cognitive functioning	76.8 ± 21.5	85.6 ± 18.8	73.6 ± 21.7	0.9	(− 4.9)–(23.1)	0.199	0.6
Social functioning	41.1 ± 33.9	46.7 ± 34.6	39.1 ± 33.9	1.5	(− 6.3)–(37.4)	0.159	0.2
Fatigue	61.1 ± 31.9	46.7 ± 33.1	66.2 ± 30.2	1.8	(− 39.7)–(1.4)	0.067	− 0.6
Nausea and vomiting	12.0 ± 17.7	7.8 ± 15.3	13.5 ± 18.5	1.8	(− 16.3)–(6.8)	0.413	− 0.3
Pain	35.6 ± 34.1	13.3 ± 29.0	43.4 ± 32.6	3.8	(− 46.5)–(− 6.4)	**0.011**	− 0.9
Dyspnea	47.4 ± 41.3	40 ± 40.2	50.0 ± 41.8	0.3	(− 37.3)–(16.6)	0.445	–0.2
Insomnia	46.8 ± 34.4	15.5 ± 30.5	57.9 ± 28.6	9.7	(− 59.8)–(− 21.6)	** < 0.001**	− 1.5
Appetite loss	33.3 ± 36.2	20 ± 32.9	38.1 ± 36.5	1.2	(− 38.5)–(8.5)	0.207	− 0.5
Constipation	27.5 ± 35.7	13.3 ± 30.4	32.5 ± 36.4	1.1	(− 37.7)–(7.3)	0.181	− 0.6
Diarrhea	16.1 ± 23.6	6.7 ± 18.7	19.4 ± 24.4	1.8	(− 27.7)–(1.6)	0.080	− 0.6
Financial difficulties	22.8 ± 32.8	11.1 ± 24.1	27.0 ± 34.7	1.4	(− 34)–(6.9)	0.190	− 0.5

*PSQI*, Pittsburgh Sleep Quality Index; *PAS*, Physical Activity Scale; *MFI*, Multidimensional Fatigue Inventory; *HADS*, Hospital Anxiety and Depression Scale; *EORTC QLQ-C30*, European Organization for Research and Treatment of Cancer – Quality of life; **Bold**, statistically significant.

HADS-Anxiety was higher than HADS-Depression. Concerning the EORTC-QOL-30, cognitive functioning was the highest functioning scale, while the fatigue scale was the most reported among symptoms (Table 2).

### Comparison between good and bad sleepers

The ANCOVA results and comparisons between good and bad sleepers are shown in Table 2. The mean value of days between the onset of chemotherapy and completion of questionnaires was 5.5 ± 3.5 days.

In general, good sleepers reported significantly better values on the physical activity, fatigue, anxiety, depression, and certain EORTC-QOL-30 subscales (emotional functioning, pain, and insomnia).

The results showed that bad sleepers spent less time in physical activity during the week. They suffered most from fatigue, anxiety, depression, and pain. Furthermore, bad sleepers reported a lower quality of life, even though without statistical significance.

### Correlation analysis

We conducted a correlation analysis of the variables relevant to the hypothesis for the mediation analysis. Table 3 shows the principal correlation analysis.

**Table 3:** Correlation analysis

	PSQI total score	PAS
PAS	− 0.420**	
MFI-General fatigue	0.328*	− 0.456**

PSQI, Pittsburgh Sleep Quality Index; PAS, Physical Activity Scale; MFI, Multidimensional Fatigue Inventory; **Bold**, statistically significant; * = *p*-value < 0.05; ^**^ = *p*-value < 0.01

Sleep was inversely correlated with physical activity and positively correlated with fatigue. Thus, patients with less impaired sleep reported higher physical activity, and patients with more sleep problems reported higher general fatigue.

Furthermore, physical activity was inversely correlated with fatigue. Thus, the higher the physical activity, the lower the general sensation of fatigue.

### Mediation analysis

Based on the results of the correlation analysis (Table 3), we built the mediation model with sleep problems (PSQI total score) as the independent variable (X), fatigue (MFI general fatigue) as the dependent variable (Y), and physical activity (PAS) as the mediator factor (M).

**Fig. 2:**
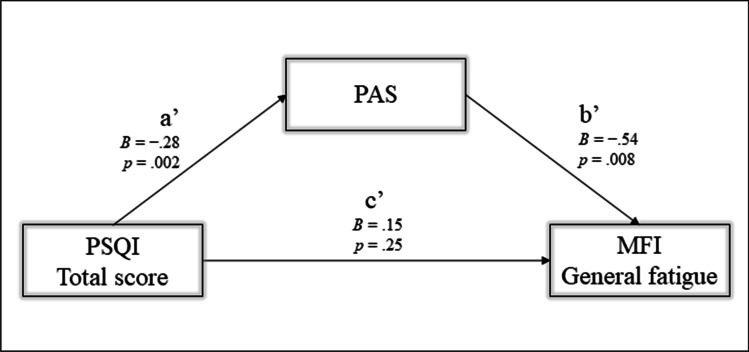
Showing the simple mediation analysis with the independent variable (PSQI Total score—X), dependent variable (MFI General Fatigue—Y), mediator (PAS—M). c’ = direct effect; a’, b’ = and indirect effects; *B* = regression coefficient; *p* = p-value

The model (Fig. 2) showed significant total effects (*B* = 0.31, *p* = 0.02) and indirect effects with significant *a’* and *b’* regression. Sleep problems (PSQI total score – X) significantly correlated with physical activity (PAS – M). The coefficient is negative, thus with a decrease in sleep problems, physical activity increased. Physical activity (PAS – M) significantly correlated with fatigue (MFI – Y). The coefficient was also negative in this case, indicating that fatigue decreased with increased physical activity. The total indirect effects, (*B* = 0.13, *BootLLCI* = 0.02, *BootULC I* = 0.35) were significant, indicating that sleep affected fatigue indirectly through physical activity.

## Discussion

To the best of our knowledge, this is the first study to provide an in-depth analysis of sleep problems and their consequences during the onset of therapy in newly-diagnosed patients with hematological cancer diseases.

Based on the cut-off levels of the PSQI, and compared to healthy sample norms [24], our results reveal that sleep problems affected a considerable proportion (ca 75%) of the study population. These findings are in line with those of previous studies that report sleep problems in comparable populations, although these studies mainly focus on later stages of the disease and its treatment (e.g., during or after medical treatment) [4, 34]. In contrast, the present study results highlight that sleep problems may occur at the onset, and, more precisely, during the first two weeks of medical therapy.

Comparing individuals classified as either “good” or “bad” sleepers provides an initial suggestion as to the potential causes of sleep problems. On the one hand, the pathology and its symptoms could exert a compromising effect on sleep. Indeed, individuals identified as “bad” sleepers reported significantly increased pain and other symptoms, even though not significant, such as nausea and vomiting, constipation, and diarrhea symptoms. Pain especially is a symptom that has already been found to interfere with sleep by causing awakening during nights [5, 35]. On the other hand, psychological distress (anxiety and depressive symptoms) due to diagnosis could negatively affect sleep as patients worry about their future and the imminent therapy. In fact, bad sleepers reported higher anxiety levels in the present study. Based on the results of previous studies, we can suppose that anxiety and depression could negatively affect sleep by increasing the difficulty of falling asleep and subsequently maintaining sleep [2, 36]. The combination of disturbed sleep, distress, anxiety, and pain could negatively impact one’s overall perception of quality of life. Indeed, bad sleepers in the present sample reported a slightly compromised perception of global health compared to good sleepers, even though the analysis showed no significance. Other factors impacting sleep and that should be taken into account are the new and unfamiliar hospital environment and daytime napping. Regarding the former, being hospitalized could represent an additional problem that could negatively affect sleep compared to day hospital care and therapy. However, due to the covered period by PSQI (30 days), sleep problems in the present sample could be mainly anxiety-induced by the disease manifestation and testing prior to the diagnosis and less to chemotherapy side effects. Focusing on daytime napping, even though we did not investigate naps habits or opportunities, based on previous results [37], we could speculate that usual naps during the day could disrupt nighttime sleep and, consequently, increasing self-perceived fatigue.

Interestingly, bad sleepers also reported decreased physical performance, physical activity levels, and increased sensation of cancer-related fatigue. Leukemia patients have previously been described as physically impaired because the higher fatigue level likely provokes tiredness, weakness, and fatigability, which may contribute to a lower level of physical activity [16]. Furthermore, specific circumstances of this patient population (for example being connected to infusion for several hours) reduce their ability to move around. Regarding physical activity, to date, studies have investigated the influence of physical activity and exercise on cancer-related fatigue in persons with cancer. In addition, sleep problems have been found to influence physical activity and physical performance. However, cancer-related fatigue has been previously defined as an independent symptom that cannot be attenuated by a sufficient amount of sleep [38]. To determine the potential interaction between these three factors, we created the mediation model based on the relevant variables identified in the correlation analysis. The mediation analysis identified physical activity in everyday life as a mediating factor between sleep and general fatigue. This indicates that sleep problems affect fatigue indirectly through physical activity. In other words, better sleep positively affects physical activity level, which in turn improves one’s sensation of fatigue. In light of previous works, it is possible to suppose that subjects would feel more inclined to be more physically active if they suffered less from sleep problems. [39, 40]. Thus, the combined action of sleep and physical activity could be a useful and effective tool to improve sensation of cancer-related fatigue during the onset of hematological cancer treatments. Our suppositions are in line with the statement that even a night of prolonged restorative rest or sleep is insufficient to reduce cancer-related fatigue [38]. Furthermore, the National Comprehensive Cancer Network recommends practicing physical activity regularly (despite the effort required to fight the effects of fatigue) [38].

Regarding fatigue, patients with sleep problems reported elevated general fatigue. Previous studies have often generalized that leukemia patients report cancer-related fatigue without considering differences in sleep problems [41], but we have demonstrated cancer-related fatigue differences based on sleep problems.

Physical activity levels are mostly assessed during hospitalization to improve patients' well-being or quality of life. In this context, baseline data are necessary to show the changes after the hospitalization or the intervention protocol, but not to describe the dissimilarities among patients during the onset of chemotherapy. Increasing physical activity will reduce the burden of cancer-related fatigue symptoms–including sleep problems–and decrease the risk of all-cause mortality [42–44]. The present study results advance the idea that the same process is applicable since the onset of therapy. In view of this, recommendations to practice more physical activity are particularly valuable because a good level of physical functioning could help to combat cancer-related fatigue and positively interact with treatment outcomes, as stated in a recent study by Möller and colleagues (2020) [45].

The results of the present study should be considered in the context of its strengths and limitations.

Its strengths comprise addressing an underinvestigated population and the administration of a well-established sleep assessment (PSQI). In studies of leukemia patients, questionnaires usually assess sleep as a component of quality of life instead of using a specific questionnaire for sleep [6, 44, 46]. Additionally, the period of assessment is a unique aspect of the present study. Early knowledge about patient conditions, such as fatigue and sleep problems, is essential because it may predict/influence therapy tolerance and prognosis [47, 48].

The significance of this study is limited by its cross-sectional design. We describe sleep problems as influencing physical activity. Although the relationship between physical activity and sleep is mostly described in this way (i.e., the former influencing the quality of the latter [12]), the bi-directional relationship between sleep and physical activity is still debated. Objective assessments for sleep and physical activity should be used in further investigations. Finally, the time frame assessment is different depending on the questionnaire. Indeed, the lack of significance could be addressed to different time frames of the questionnaires (e.g. 30 days for the PSQI and one week for EORTC QLQ-C30). Notwithstanding the difficulties in finding appropriate methods for this population and questionnaires investigating the identical timeframe, we could speculate that the result could be even bigger and significant with a perfect timeframe overlap.

In conclusion, this study identified sleep problems, which may be driven by distress and other symptoms (e.g., anxiety and pain), as an important issue in hematological patients during the onset of the therapy.

Sleep problems represent an issue that could leave subjects with lower energy reserves (preventing physical activity) and ultimately result in cancer-related fatigue. Besides physical activity, sleep problems should be considered as a confounding factor and toehold in further studies investigating cancer-related fatigue in persons with hematological diseases and cancer. Future studies should consider more and other influencing factors linked to sleep, physical activity, and sleep, such as weight and BMI. Indeed, body composition parameters and nutritional status (even though not significant in the present study) are known to interact with sleep disturbances [49, 50]. Furthermore, upcoming studies should consider investigating sleep more broadly and also in its biological components and objective parameters. For example, cortisol should be taken into account because its low level could interact with fatigue and cytokines production. Moreover, both cortisol and melatonin, the hormone predisposing to sleep, show a circadian rhythm and their altered rhythmicity could predispose to the pathology or be affected by cancer treatments [51]. In this view, rest-activity circadian rhythm analysis and cortisol and melatonin 24-h dosages could help describe sleep problems and their causes or consequences.

## Data Availability

N/A

## Code availability

N/A
